# Beyond Lipid Lowering: A Narrative Review and Expert Perspective on Precision Cardiovascular Prevention in People with HIV After REPRIEVE

**DOI:** 10.3390/biom16091254

**Published:** 2026-08-29

**Authors:** Pere Domingo, Paula Prieto

**Affiliations:** 1 Malalties Infeccioses, Hospital de la Santa Creu i Sant Pau, Sant Quintí, 41-49, 08041 Barcelona, Spain; pprietox@santpau.cat; 2 Institut de Recerca del Hospital de la Santa Creu i Sant Pau, Reial Acadèmia de Medicina de Catalunya, 08001 Barcelona, Spain; 3 Departament de Medicina, Universitat Autònoma de Barcelona, 08023 Barcelona, Spain

**Keywords:** HIV, cardiovascular disease, atherosclerosis, statins, inflammation, biomarkers, immunometabolism, precision medicine, REPRIEVE

## Abstract

The spectrum of diseases in individuals with human immunodeficiency virus (HIV) receiving successful antiretroviral therapy has evolved over time. In the past, they developed opportunistic infections and malignancies, whereas today, cardiovascular disease is among the most common causes of illness and premature death. Traditional risk factors for atherosclerosis (hypertension, hyperlipidemia, smoking, diabetes, family history of heart disease) are more prevalent in people with HIV than in the general population. However, it is well established that HIV itself causes increased immune activation, chronic inflammation, vascular dysfunction, and a cluster of metabolic abnormalities that contribute to a faster-than-usual rate of biological aging and a higher risk of developing atherosclerosis, a risk not fully captured by current risk models. In the REPRIEVE study, treatment with pitavastatin was shown to reduce the rate of first cardiovascular events among individuals with HIV receiving antiretroviral therapy. Importantly, the beneficial effects of statins on atherosclerosis likely extend beyond lowering cholesterol to include effects on vascular function and on immune and metabolic systems altered by HIV. Even among individuals on statins, a considerable risk of cardiovascular disease remains. Here, We provide a narrative review of current evidence and an expert perspective on emerging approaches to residual cardiovascular risk after REPRIEVE. We review the current understanding of atherosclerosis pathogenesis in individuals with HIV, focusing on recent findings from the REPRIEVE trial. We outline current approaches to improving cardiovascular risk assessment across clinical, biological, and computational levels. We also examine a growing number of therapeutic options that address residual inflammation and atherogenic metabolic disturbance in individuals with HIV on long-term, effective antiretroviral therapy. Significantly, after REPRIEVE, we must move from prescribing statins to all individuals with HIV toward more individualized cardiovascular disease prevention strategies, integrating clinical information, a variety of biomarkers, imaging studies, and even molecular information to generate optimal individualized cardiovascular disease prevention regimens that reflect the complexity of this outcome in naturally diverse individuals.

## 1. Introduction

Effective antiretroviral therapy (ART) has transformed HIV into a chronic condition, and life expectancy among many treated people with HIV (PWH) now approaches that of the general population [1,2,3]. Consequently, HIV care increasingly focuses on non-communicable diseases, particularly atherosclerotic cardiovascular disease (ASCVD) among individuals with controlled viremia [4,5].

PWH have a 1.5- to 2-fold higher risk of myocardial infarction, stroke, heart failure, and cardiovascular death than people without HIV, even after adjusting for conventional risk factors [6,7]. This excess risk reflects both the higher prevalence of smoking, hypertension, dyslipidemia, and diabetes and persistent HIV-related mechanisms, including immune activation, chronic inflammation, microbial translocation, vascular dysfunction, oxidative stress, coagulopathy, metabolic disturbance, and accelerated biological aging [8,9,10,11].

Despite effective ART, residual viral activity, gut-barrier dysfunction, opportunistic or chronic co-infections, and comorbidities can sustain inflammation and immune activation [8,10,11]. Both innate and adaptive immune pathways contribute to atherogenesis [8,10,12], and metabolic dysregulation and immune aging may further amplify risk [11,12]. These processes may help explain the severe, multifocal, lipid-rich, and potentially vulnerable plaque phenotype observed in PWH [5,13].

REPRIEVE was a landmark international trial of 7769 adults with HIV receiving ART, aged 40–75 years and at low-to-moderate estimated 10-year cardiovascular risk. Pitavastatin reduced major cardiovascular events by 36% [14,15,16]. Its mechanistic substudy also showed slower progression of non-calcified coronary plaque, consistent with effects beyond lipid lowering [17,18]. These findings led to updated guidelines recommending statin therapy as part of primary prevention for PWH who meet eligibility criteria [19,20,21,22].

Nevertheless, substantial residual cardiovascular risk persists, and conventional calculators may underestimate risk in PWH and in groups underrepresented in their derivation [23,24]. Biomarkers, advanced imaging, machine learning, and multi-omics may eventually refine risk stratification, but their clinical value remains investigational and requires prospective validation [25].

This narrative review examines the molecular basis of HIV-associated atherosclerosis, the immune and metabolic effects of statins, and the clinical implications of REPRIEVE. It also critically appraises residual cardiovascular risk and emerging biomarker, imaging, computational, and therapeutic approaches, distinguishing established evidence from expert interpretation and outlining future research priorities.

## 2. Review Methodology

This article is a narrative review that incorporates expert perspectives and was not conducted as a systematic review or meta-analysis. Relevant literature was identified through targeted searches of PubMed/MEDLINE and by examining the reference lists of key publications through July 2026. Search concepts included HIV, atherosclerosis, cardiovascular disease, cardiovascular risk, statins, pitavastatin, REPRIEVE, inflammation, immune activation, immunometabolism, biomarkers, cardiovascular imaging, coronary computed tomography angiography, multi-omics, artificial intelligence, and precision prevention.

Priority was given to randomized clinical trials, major prospective and retrospective cohort studies, systematic reviews and meta-analyses, scientific statements, clinical guidelines, and recent mechanistic studies relevant to HIV-associated cardiovascular disease. Particular emphasis was placed on the REPRIEVE trial and its mechanistic and imaging substudies, with evidence from the general cardiovascular population included when directly relevant to biological mechanisms or potential future interventions in people with HIV.

Study identification and selection were guided by the authors’ expertise and judgment regarding relevance, methodological quality, and contribution to the review’s conceptual framework. No prespecified protocol, formal study-screening process, risk-of-bias assessment, or quantitative synthesis was conducted. Accordingly, the article should be interpreted as a narrative synthesis of current evidence, combined with expert interpretation. Future-oriented concepts, including artificial-intelligence-assisted risk prediction, multi-omics integration, and precision cardiovascular prevention, are presented as investigational strategies requiring prospective validation rather than as established conclusions of REPRIEVE or current standards of care.

## 3. Molecular Basis of HIV-Associated Atherosclerosis

**Figure 1:**
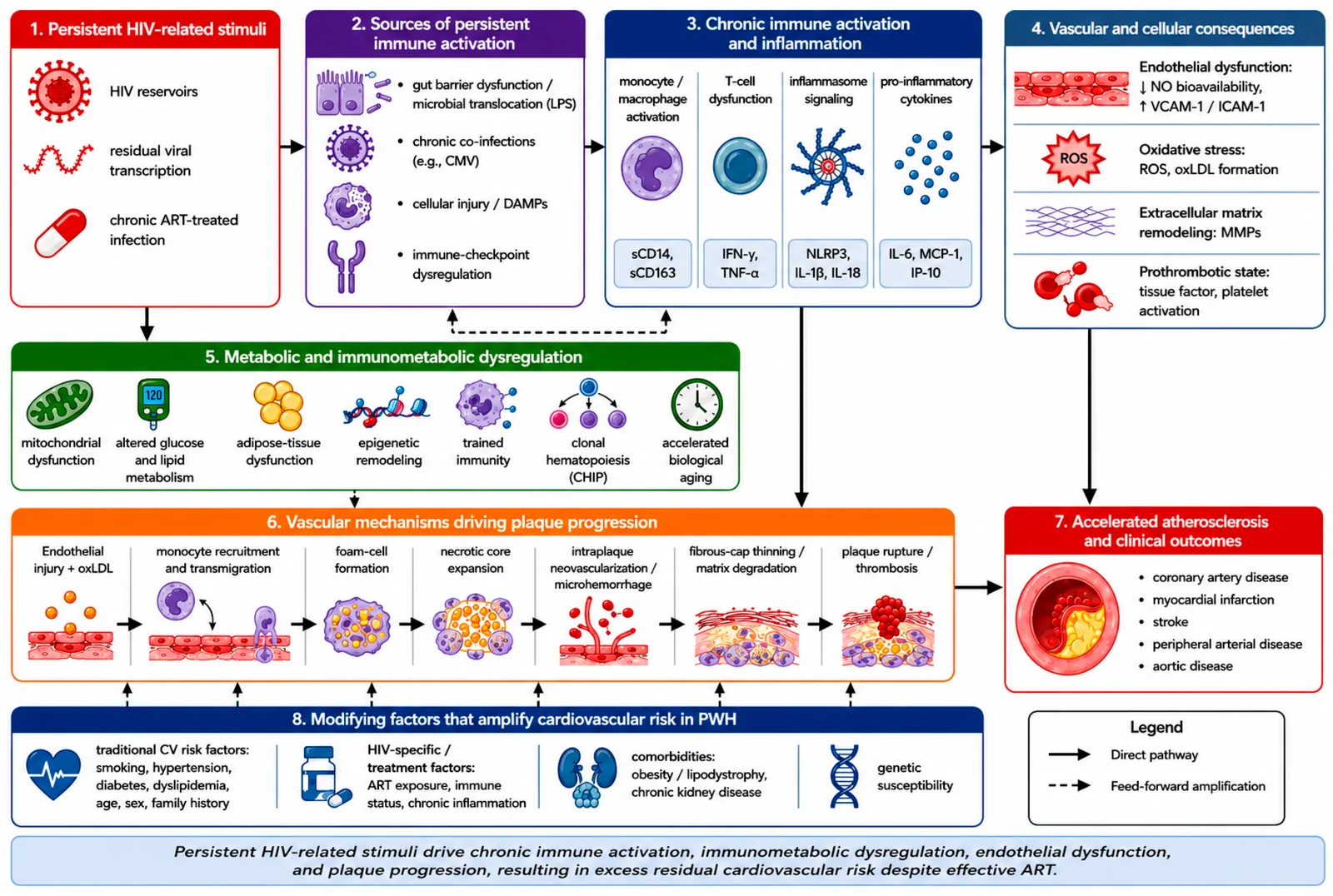
Mechanistic hierarchy of HIV-associated atherosclerosis and residual cardiovascular risk. Persistent HIV infection drives a mechanistic hierarchy of interconnected biological events that culminates in accelerated atherosclerosis and excess residual cardiovascular risk despite effective antiretroviral therapy. The figure illustrates the progression from persistent HIV-related stimuli to chronic immune activation, immunometabolic dysregulation, vascular injury, plaque progression, and clinical cardiovascular disease, while emphasizing the multiple feed-forward interactions that amplify these processes. Persistent HIV-related stimuli—including viral reservoirs, residual viral transcription, and chronic infection despite ART—sustain continuous immune stimulation through microbial translocation due to gut barrier dysfunction, chronic viral co-infections, cellular injury, and immune checkpoint dysregulation. These factors drive chronic activation of monocytes/macrophages and T cells, inflammasome signaling, and sustained production of pro-inflammatory cytokines such as interleukin (IL)-6, monocyte chemoattractant protein-1 (MCP-1), interferon-γ-induced protein 10 (IP-10), tumor necrosis factor-α (TNF-α), and interferon-γ (IFN-γ), along with increased expression of soluble CD14 (sCD14) and soluble CD163 (sCD163). Chronic immune activation is accompanied by profound metabolic and immunometabolic dysregulation, characterized by mitochondrial dysfunction, altered glucose and lipid metabolism, adipose tissue dysfunction, epigenetic remodeling, trained immunity, clonal hematopoiesis of indeterminate potential (CHIP), and accelerated biological aging. These mechanisms interact with endothelial dysfunction, oxidative stress, extracellular matrix remodeling, and a prothrombotic state to promote oxidation of low-density lipoprotein cholesterol (oxLDL), monocyte recruitment, foam cell formation, necrotic core expansion, intraplaque neovascularization, fibrous cap degradation, plaque rupture, and thrombosis. Traditional cardiovascular risk factors, HIV-specific characteristics, cumulative ART exposure, chronic inflammation, metabolic comorbidities, and genetic susceptibility further amplify these pathogenic pathways, leading to earlier onset, greater plaque burden, and increased plaque vulnerability in people with HIV (PWH). Together, these interconnected mechanisms provide the biological basis for the persistent excess cardiovascular risk observed in PWH despite effective viral suppression and support the development of therapeutic strategies targeting inflammation, immunometabolism, and residual cardiovascular risk. Abbreviations: ART, antiretroviral therapy; CHIP, clonal hematopoiesis of indeterminate potential; CMV, cytomegalovirus; DAMPs, damage-associated molecular patterns; IFN-γ, interferon-gamma; IL, interleukin; IP-10, interferon-γ-induced protein 10; LPS, lipopolysaccharide; MCP-1, monocyte chemoattractant protein-1; MMPs, matrix metalloproteinases; NO, nitric oxide; oxLDL, oxidized low-density lipoprotein; PWH, people with HIV; ROS, reactive oxygen species; sCD14, soluble CD14; sCD163, soluble CD163; TNF-α, tumour necrosis factor-alpha; VCAM-1, vascular cell adhesion molecule-1.

Atherosclerosis in PWH is believed to result from a combination of traditional CVD factors and persistent HIV-related biological mechanisms, even with long-term effective ART. There is growing evidence that the nature and risk of CVD in PWH differ from and may exceed those of their uninfected counterparts (Figure 1). Furthermore, traditional CVD scoring systems have limited accuracy in predicting risk among PWH [4,10,11,12,26,27].

Chronic immune activation is thought to be the primary mechanism driving the increased risk of atherosclerosis in PWH. Several mechanisms are believed to contribute to this chronic activation, including persistent HIV replication, microbial translocation due to ongoing gastrointestinal damage, other persistent infections (e.g., cytomegalovirus), immune senescence, and dysregulation of both innate and adaptive immune responses [4,11,26]. Activated monocytes, macrophages, dendritic cells, T cells of the adaptive immune system, and vascular endothelial cells all produce large amounts of proinflammatory cytokines and chemokines, as well as reactive oxygen species (ROS) and procoagulant substances, as illustrated in Figure 1. The earliest event in the pathogenesis of atherosclerosis in PWH involves the activation of circulating monocytes, which then migrate into the arterial intima [11,28]. Upon activation, monocytes that have entered the arterial intima differentiate into macrophages. Macrophages within atherosclerotic plaques are the principal cell type that takes up modified lipids, including oxidized low-density lipoprotein (oxLDL), thereby forming foam cells [28]. Moreover, activated macrophages, such as those that have entered the arterial intima, produce a variety of bioactive substances, including cytokines, growth factors, and matrix metalloproteases, and can also express tissue factor, thereby fostering atherosclerosis. In addition to cytokines produced by activated macrophages in the arterial intima, cytokines produced by other activated cells contribute to inflammation in the intima [5,11]. In PWH, circulating sCD163 has been associated with non-calcified coronary plaque [29,30], whereas sCD14 has been associated with coronary calcification and the extent of subclinical vascular disease [31]. Elevated sCD14 has also been associated with mortality, although it is not a plaque-specific biomarker [32]. Thus, activated monocytes/macrophages play a crucial role in the pathogenesis of atherosclerosis in PWH.

Another component of the adaptive immune response that contributes to atherosclerosis is T-cell dysfunction. Activation of CD4^+^ and CD8^+^ T cells induces the production of interferon-γ (IFN-γ), tumor necrosis factor-α (TNF-α), and other proinflammatory cytokines, including interleukin-6 (IL-6), which leads to endothelial activation and the subsequent recruitment of leukocytes to sites of atherosclerosis. Additionally, dysfunction of CD4^+^ Tregs and other Treg subsets, as well as T-cell immune senescence, contributes to chronic vascular inflammation [8,11].

Endothelial dysfunction is an early marker of HIV-associated vascular disease and plays a critical role in the pathogenesis of atherosclerosis in PWH. It reduces nitric oxide bioavailability and increases reactive oxygen species (ROS) production, which promote leukocyte adhesion and transmigration across the endothelium by upregulating vascular cell adhesion molecule-1 (VCAM-1), intercellular adhesion molecule-1 (ICAM-1), and E-selectin [10]. Oxidative stress from activated immune cells, along with mitochondrial dysfunction, oxidizes low-density lipoprotein cholesterol (LDL-C), which is taken up by macrophages within the arterial wall, forming foam cells that are the hallmark of atherosclerotic plaque [28]. The biochemistry of atherosclerotic plaque in PWH is characterized by the early formation of large, lipid-rich, non-calcified plaques that are prone to rupture [33].

Recent studies have established that immunometabolic reprogramming of innate immune cells contributes to the pathogenesis of HIV-associated vascular disease by increasing glycolysis, altering mitochondrial function, shifting fatty acid oxidation, and activating the mevalonate pathway to sustain chronic inflammatory signaling. Innate immune cells previously exposed to pathogens can retain a pro-inflammatory phenotype, known as trained immunity [34], which contributes to persistent inflammation in PWH on effective ART and to increased CVD that cannot be explained by conventional clinical risk factors, even after mitigation of these factors [11,30,32]. Inflammasome activation has emerged as a mechanism linking HIV infection to accelerated CVD. The NLRP3 inflammasome is a multiprotein complex that regulates the production of interleukin-1β (IL-1β) and interleukin-18 (IL-18), two cytokines with potent pro-inflammatory and proatherogenic effects. Activation of the NLRP3 pathway has been implicated in both HIV persistence and atherosclerosis [28,30]. Although trained immunity provides a compelling mechanistic framework, it is not currently measured in routine clinical practice, and no validated trained-immunity biomarker or treatment algorithm is available for cardiovascular prevention in PWH.

Aging is another biological process associated with increased CVD in PWH. Chronological age reflects elapsed time, whereas biological age aims to capture interindividual differences in physiological and molecular decline. Proposed measures include epigenetic clocks, telomere length, cellular-senescence markers, mitochondrial dysfunction, and composite biomarker indices. However, these measures are not interchangeable; their validation and reproducibility vary, and none is currently established for routine CVD stratification or treatment selection in PWH. Chronic HIV infection accelerates biological aging through mechanisms that include telomere shortening, mitochondrial dysfunction, cellular senescence, and epigenetic alterations [35]. These processes culminate in premature vascular aging, marked by increased arterial stiffness, endothelial dysfunction, and heightened susceptibility to atherosclerotic disease. These factors may lead to significant variability in CVD among individuals of the same age. As a result, conventional risk scoring systems are limited in predicting CVD in individuals with HIV [36,37]. Biological-age measures may help characterize heterogeneity in cardiovascular vulnerability, but their clinical utility for risk classification or treatment selection in PWH remains unproven.

Collectively, these processes can explain the specific characteristics of atherosclerosis in PWH, including earlier onset and a higher proportion of noncalcified, high-risk plaques. Importantly, many of these processes can still increase CVD even when HIV is suppressed and are not captured by traditional risk scoring systems developed for the general population.

## 4. Statins in PWH: Biological Basis and Clinical Evidence After REPRIEVE

**Figure 2:**
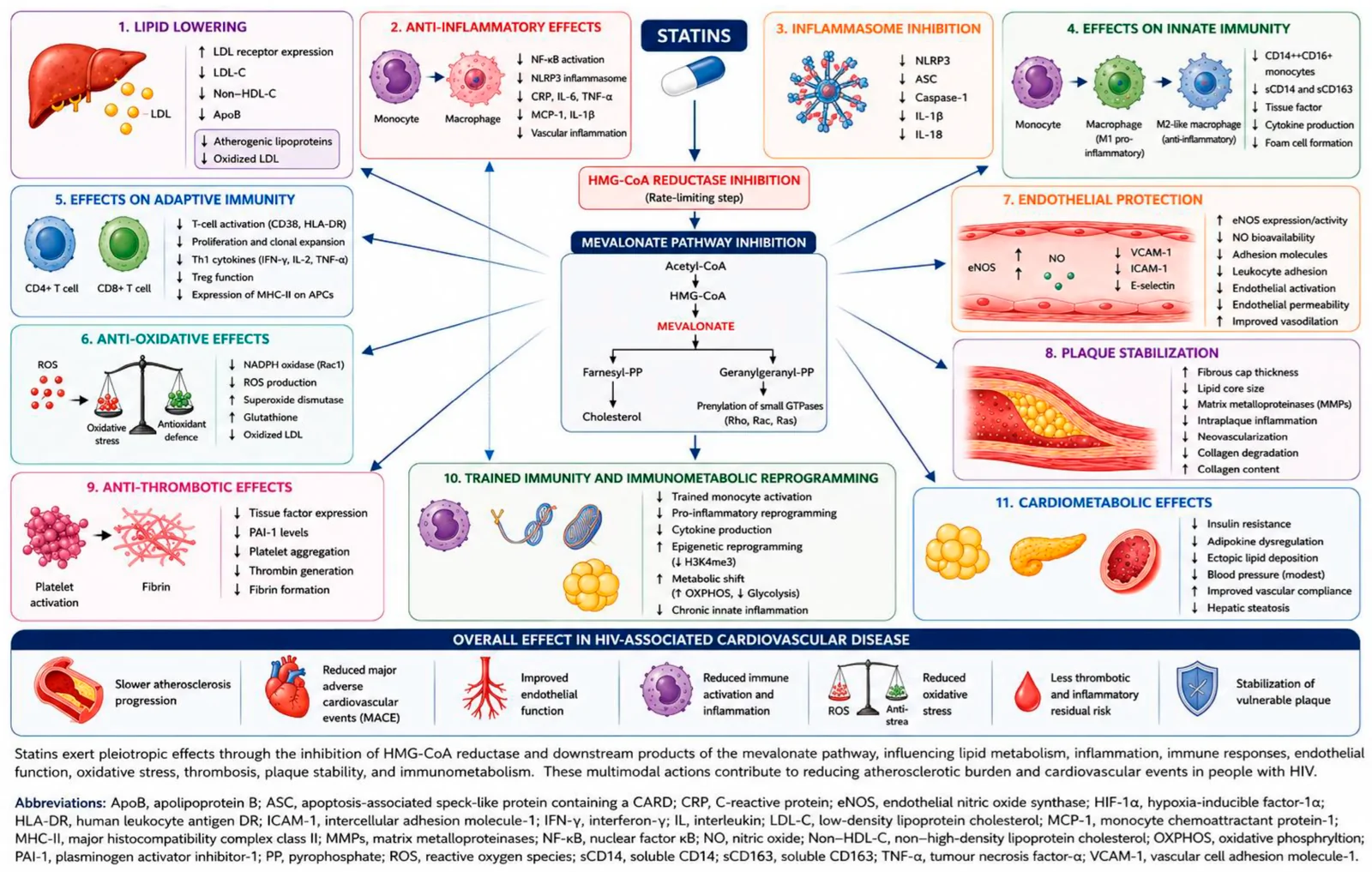
Mechanisms by which statins modulate HIV-associated atherosclerosis and residual cardiovascular risk. Statins reduce CVD in PWH through multiple complementary mechanisms that extend far beyond lowering LDL-C. Inhibiting HMG-CoA reductase suppresses the mevalonate pathway, thereby modifying inflammatory, vascular, metabolic, thrombotic, and immunometabolic processes that contribute to HIV-associated atherosclerosis. Beyond lowering circulating LDL-C, non-HDL-C, and ApoB, statins attenuate NF-κB activation, inhibit the NLRP3 inflammasome, reduce production of IL-1β, IL-6, TNF-α, and MCP-1, and reduce vascular inflammation. They also modulate innate and adaptive immune responses by decreasing monocyte activation, promoting macrophage polarization toward a less inflammatory phenotype, reducing sCD14 and sCD163, attenuating T-cell activation, and improving regulatory T-cell function. Statins improve endothelial function by increasing eNOS activity and nitric oxide bioavailability while reducing endothelial activation, oxidative stress, leukocyte recruitment, endothelial permeability, and the expression of vascular adhesion molecules. Within the arterial wall, these effects promote plaque stabilization by reducing lipid-core size, matrix metalloproteinase activity, intraplaque inflammation, neovascularization, and thrombotic activation, while increasing fibrous cap thickness and collagen content. Statins modulate trained immunity by inhibiting mevalonate-dependent metabolic and epigenetic reprogramming of innate immune cells, thereby attenuating persistent inflammation despite suppressive ART.

(3-Hydroxy-3-methylglutaryl-coenzyme A) HMG-CoA reductase inhibitors, or statins, have traditionally been viewed as simple lipid-lowering agents that reduce CVD, in part by lowering LDL-C [38,39,40]. However, it has become clear that statins have a broad array of additional biologic actions. Many of these actions are likely to reduce the risk of atherosclerosis and its complications, including in PWH, in whom atherosclerosis is an increasingly recognized cause of suboptimal outcomes. This review will describe the pleiotropic actions of statins (Figure 2) and present data from clinical trials in PWH in whom statins have been shown to reduce the risk of a wide variety of adverse cardiovascular events.

Beyond LDL-C lowering, statins inhibit the mevalonate pathway and downstream Rho, Rac, and Ras signaling [38,39]. These effects improve nitric oxide bioavailability and reduce oxidative stress, endothelial activation, leukocyte recruitment, and inflammatory signaling and may attenuate mevalonate-dependent trained immunity. Statins may therefore act as immunometabolic modulators in addition to their established lipid-lowering effects [38,39,40].

Before the REPRIEVE trial, studies using a variety of designs to investigate PWH found that, compared with placebo, statins had a range of beneficial effects, including decreases in biomarkers of inflammation and immune activation, measures of monocyte activation, and subclinical atherosclerosis as assessed by non-invasive imaging, as well as improvements in endothelial function. In addition, reports indicated a slower rate of increase in non-calcified coronary plaque in PWH treated with statins compared with those receiving placebo [13,41,42,43].

The REPRIEVE trial was a randomized, double-masked, placebo-controlled trial of pitavastatin in 7769 PWH aged 40 to 75 years on ART with low-to-moderate estimated CVD, with a median follow-up of 5.1 years. Pitavastatin reduced MACE by 36% (hazard ratio, 0.64; 95% CI, 0.48–0.84), with an incidence-rate difference of approximately 1.3 events per 1000 person-years [15,16]. There are several key points to remember about the REPRIEVE trial. First, the trial demonstrated that PWH who are on effective ART and have a low-to-moderate estimated risk of CVD would benefit from a statin. In other words, the trial showed that PWH would benefit from statin therapy, even when traditional risk assessment tools do not indicate increased CVD. In this regard, the trial highlights important limitations of currently used CVD assessment models in PWH. The broad biological actions illustrated in Figure 2 provide the mechanistic framework supporting the clinical observations reported in the REPRIEVE trial.

REPRIEVE provides high-quality evidence that pitavastatin reduces major adverse cardiovascular events in PWH receiving ART who are 40–75 years of age and have low-to-moderate estimated cardiovascular risk. However, both relative and absolute treatment effects should be considered. Pitavastatin produced a 36% relative reduction in MACE, but the underlying event rate was low, as expected in a primary-prevention population. Accordingly, the absolute reduction was more modest, and the number needed to treat varied substantially by baseline CVD, becoming more favorable among participants with higher predicted risk [15,16].

The trial’s external validity also requires careful interpretation. REPRIEVE was geographically diverse and included substantial representation of women and participants from low- and middle-income countries, strengthening its global relevance. Nevertheless, eligibility criteria selected adults receiving ART with relatively preserved renal and hepatic function and without known cardiovascular disease or conventional indications for statin therapy. Therefore, the findings cannot be directly extrapolated to people younger than 40 years or older than 75 years, those not receiving ART, individuals with uncontrolled HIV replication, people with established ASCVD, or those at very high or extremely low baseline cardiovascular risk. In these groups, treatment decisions should remain guided by available general-population recommendations, HIV-specific risk enhancers, drug–drug interactions, comorbidities, and shared decision-making.

Implementation may also vary across healthcare systems. The clinical value and cost-effectiveness of broad statin use depend on baseline cardiovascular event rates, medication cost and availability, laboratory and clinical monitoring capacity, adherence, competing causes of morbidity and mortality, and the opportunity costs of long-term preventive treatment. These considerations are especially important in resource-limited settings, where the absolute cardiovascular benefit may differ from that observed in higher-income regions and where generic pitavastatin may not be widely available. Therefore, formal country-specific economic analyses and implementation studies are needed before assuming that a single preventive strategy is optimal across all settings [44,45].

Thus, REPRIEVE establishes pitavastatin as an effective primary-prevention intervention for the population studied, but it should not be interpreted as evidence for indiscriminate statin treatment of all PWH. Instead, its findings should be integrated with baseline absolute risk, patient preferences, treatment burden, local resources, and current guideline recommendations.

The magnitude of cardiovascular benefit observed in REPRIEVE, together with findings from its mechanistic substudy, is consistent with effects extending beyond LDL-C lowering alone. However, the relative contributions of lipid lowering, anti-inflammatory activity, and other immunomodulatory mechanisms cannot be determined directly from the trial [17,18]. Previous studies in PWH have reported favorable effects of statins on monocyte activation, endothelial function, oxidative stress, oxidized lipids, and coronary plaque characteristics [17,33,41,42,43,46,47]. In experimental and general-population studies, statins improve endothelial nitric oxide bioavailability and reduce endothelial activation and oxidative stress [38,39,40]. Statins reduce oxidative stress by inhibiting the Rac1 pathway, which regulates NADPH oxidase in vascular smooth muscle and endothelial cells, thereby reducing ROS production [38]. In addition, they increase the production of endothelial-derived nitric oxide. A systematic review and meta-analysis of randomized trials found that high-intensity statin therapy reduced circulating oxLDL concentrations compared with low- to moderate-intensity therapy. In PWH, oxidized lipids have been associated with monocyte activation and vascular disease, and their reduction during statin therapy has correlated with improvements in these processes [33,47]. Finally, the pro-inflammatory reprogramming of monocytes and macrophages induced by mevalonate is reversed by statin inhibition of HMG-CoA reductase [48].

In terms of safety, the results of the REPRIEVE trial provide strong evidence that pitavastatin is safe for use in PWH, with only a modest increase in muscle complaints and newly diagnosed diabetes, comparable to those in the general population receiving statin therapy [49,50] and with few clinically meaningful drug interactions with ART. This lack of interactions is expected, given that pitavastatin is neither a substrate for nor an inhibitor of cytochrome P450 enzymes [21].

Further analysis of the REPRIEVE trial has confirmed pitavastatin’s cardiovascular benefits. Among 751 participants who underwent coronary computed tomography angiography (CCTA) at study entry and again at the end of the trial, progression of non-calcified coronary plaque volume was lower with pitavastatin than with placebo, consistent with a favorable effect on coronary plaque burden [17]. Clinical implementation should be tailored to local resources and healthcare priorities. Although statins are generally feasible, costs, availability, drug monitoring, adherence, and competing health needs vary substantially across settings. Advanced imaging, biomarker panels, multi-omics, and AI-based tools remain costly and inaccessible in many regions, potentially widening existing disparities. In low- and middle-income countries, implementation should therefore prioritize affordable, evidence-based measures, including smoking cessation, blood pressure and diabetes control, and access to appropriate statin therapy, while more complex approaches should be validated locally and assessed for cost-effectiveness.

Cardiovascular benefit in PWH may occur in a subset of individuals. It may be greatest in those with higher levels of inflammation, a high risk of CVD, or extensive coronary plaque, as identified by current biomarkers and imaging techniques. Residual risk of CVD in PWH on effective ART likely reflects ongoing immune activation and other mechanisms of biological aging, as well as modifiable risk factors such as obesity, elevated lipoprotein(a), CHIP, and immunometabolic dysregulation. Thus, the findings of the REPRIEVE trial will serve as a foundation for future studies aimed at preventing CVD in PWH and will require implementing precision prevention strategies that account for an individual’s unique combination of clinical, imaging, and biochemical characteristics. Therefore, the REPRIEVE trial should not be used as evidence that all PWH should be on a statin, since its results cannot be generalized to all adult PWH, including children, older adults, PWH not on ART, or those with very high or very low estimated CVD. Rather, the REPRIEVE trial must serve as the starting point for a more refined approach to preventing CVD in adult PWH. REPRIEVE established pitavastatin as an effective primary-prevention intervention for adults with HIV who resembled the trial population, while leaving uncertainty for groups outside its eligibility criteria. The trial’s results also clearly indicate that a significant residual risk of cardiovascular events remains among individuals receiving statins and that new approaches to preventing CVD are needed. Since current risk assessment tools do not capture many variables that may influence CVD in PWH, a more precision-oriented approach to prevention is warranted.

## 5. Residual Cardiovascular Risk: From Biomarkers to Precision Risk Stratification

The REPRIEVE trial showed that in PWH on ART, initiating pitavastatin reduced the risk of cardiovascular events and established statins as a cornerstone of primary prevention of cardiovascular disease in this population. Despite effective lipid lowering, a significant residual risk of cardiovascular events remained [14,16]. This residual risk is thought to be driven by a multitude of factors, including chronic immune activation, ongoing inflammation, an imbalance in metabolic pathways, and other factors affecting the cardiovascular system, such as vascular aging and characteristics of atherosclerotic plaque. The next frontier in the cardiovascular health of PWH will therefore be identifying those at the highest residual risk of cardiovascular events. The approaches discussed below should be interpreted as emerging research strategies rather than established standards of care. REPRIEVE demonstrated the benefit of pitavastatin in its enrolled population but did not test biomarker-guided treatment, multi-omics-based prevention, artificial-intelligence-supported risk stratification, or individualized therapeutic algorithms.

Precision cardiovascular prevention should be understood as a spectrum rather than as a single established clinical strategy. In current practice, it refers to tailoring evidence-based prevention to the characteristics of the individual patient. This includes assessment of conventional CVD factors, HIV-specific risk enhancers, immunological history, ART exposure, comorbidities, drug–drug interactions, and patient preferences. It also includes individualized decisions regarding statin initiation and intensity, smoking cessation, blood pressure and diabetes management, weight control, and the selective use of established imaging tests when their results are likely to modify management [5,19,20,21,22,23,24].

By contrast, a more advanced form of precision prevention aims to integrate circulating biomarkers, advanced plaque imaging, biological-age measures, clonal hematopoiesis, multi-omics data, and artificial-intelligence-based prediction models [25,51,52,53,54,55,56,57,58,59,60,61]. These approaches may eventually help identify distinct biological pathways of residual CVD and guide targeted interventions. However, most have not been prospectively validated as integrated clinical decision tools in PWH, and their incremental value beyond conventional risk assessment remains uncertain [23,24,25,59,60,61].

Accordingly, the term “precision cardiovascular prevention” is used in this review in two distinct ways: first, to describe the individualized application of currently established preventive measures; and second, to describe an investigational framework that may, in the future, incorporate biological, imaging, and computational data. The latter should be regarded as hypothesis-generating and should not be interpreted as a current standard of care.

For clarity, the approaches discussed in this review are categorized into three levels of evidence. Established clinical evidence comprises interventions supported by randomized trials, guidelines, or accepted clinical practice. Promising translational approaches include biomarkers, biological age measures, trained immunity signatures, CHIP assessment, and advanced imaging techniques that have shown biological or prognostic associations but are not yet validated for routine treatment selection. Speculative future applications include integrated multi-omics platforms, artificial-intelligence-based decision tools, and biomarker-guided therapeutic algorithms, which remain hypothesis-generating and require prospective validation.

Consistent with the mechanisms described above, inflammatory and immune-activation biomarkers frequently remain elevated despite suppressive ART and have been associated with subclinical atherosclerosis and cardiovascular events [11,41]. There is growing evidence that an integrated panel of biomarkers measuring inflammation, immune activation, coagulation, and metabolic pathways could improve CVD prediction [10,11]. The Canakinumab Anti-inflammatory Thrombosis Outcomes Study (CANTOS), a secondary prevention trial in a population with CVD, demonstrated that targeting inflammation, independent of cholesterol lowering, led to a significant reduction in cardiovascular endpoints [62]. Therefore, CANTOS provides strong proof of concept that, in addition to lowering lipid levels with statins, residual inflammatory risk could be an additional target for future prevention strategies in PWH.

Persistent immunometabolic dysfunction and trained-immunity phenotypes may contribute to residual vascular risk despite viral suppression. At present, however, these pathways remain primarily mechanistic and translational targets rather than validated clinical tools [48,63]. These changes are thought to sustain inflammation despite effective suppression of HIV replication and to contribute to CVD in individuals on effective ART.

In addition to the well-established variability in HIV-related CVD factors, an increasing body of evidence suggests that, in many individuals, biological age exceeds chronological age [36,37]. This will result in substantial interindividual variability in immune activation levels, the degree of endothelial dysfunction, the extent of atherosclerotic plaque, and the risk of cardiovascular events among PWH on effective ART with similar clinical characteristics [36,37]. Therefore, biological markers may eventually contribute to more individualized cardiovascular prevention, although their incremental clinical value and optimal use remain to be established.

Several emerging biomarkers are under investigation to further define cardiovascular risk in PWH. Lipoprotein(a) [Lp(a)] is among the most studied. As an atherogenic, proinflammatory, and prothrombotic factor, Lp(a) has been established as an independent risk factor for CVD. Although data on the relationship between HIV infection and Lp(a) are limited, Lp(a) remains a relevant risk factor in PWH whose CVD has not been optimally reduced with lipid-lowering therapy [4,5]. Lp(a) is emerging as a potential therapeutic target, particularly with the development of novel RNA-based therapies to lower Lp(a) levels [64,65,66,67]. At present, these biomarkers are primarily research tools. Their incremental value beyond conventional risk assessment, optimal thresholds, reproducibility, and ability to improve clinical outcomes has not been sufficiently established to support routine use.

A new category of CVD factors that has been studied in greater detail in PWH includes markers of CHIP [51,52]. In the general population, CHIP is associated with atherosclerotic cardiovascular disease, particularly when clone size is large [52,53,54,55]. In REPRIEVE, the association between CHIP and cardiovascular events was not uniform but appeared to be concentrated among participants with larger clone burdens, particularly DNMT3A-associated clones [56]. Analyses from the REPRIEVE trial suggest that CHIP is not uniformly associated with CVD in PWH. Rather, it appears to be driven by clone burden, particularly in clones involving DNMT3A, identifying a small subgroup at substantially increased risk of MACE [56]. Although CHIP may identify a subgroup with increased CVD, routine CHIP testing for cardiovascular prevention in PWH is not currently established, and no prospective evidence shows that CHIP-guided treatment improves outcomes.

Obesity and abnormal body fat distribution have also been implicated in CVD among PWH. Visceral fat accumulation, ectopic fat, and the resulting abnormal production of adipokines lead to insulin resistance, endothelial dysfunction, and chronic low-grade inflammation [68,69]. These factors have been shown to interact with the chronic immune activation in PWH. Novel therapeutic strategies to reduce CVD are being explored, including agents that target adipose tissue, such as glucagon-like peptide-1 receptor agonists (GLP-1-RAs). Large cardiovascular outcome trials have demonstrated that semaglutide reduces major cardiovascular events in people with type 2 diabetes and in people with overweight or obesity and established cardiovascular disease [70,71]. Semaglutide also produces substantial weight loss, while effects on inflammation and glycemic progression have been reported in additional analyses and mechanistic studies. GLP-1RAs also influence several pathways implicated in HIV-associated CVD, including inflammation, insulin resistance, endothelial dysfunction, epigenetic age, and adipose tissue biology [72,73,74]. Although evidence in PWH remains limited [74], ongoing studies suggest that GLP-1RAs may play a significant role in future cardiovascular prevention strategies.

**Figure 3:**
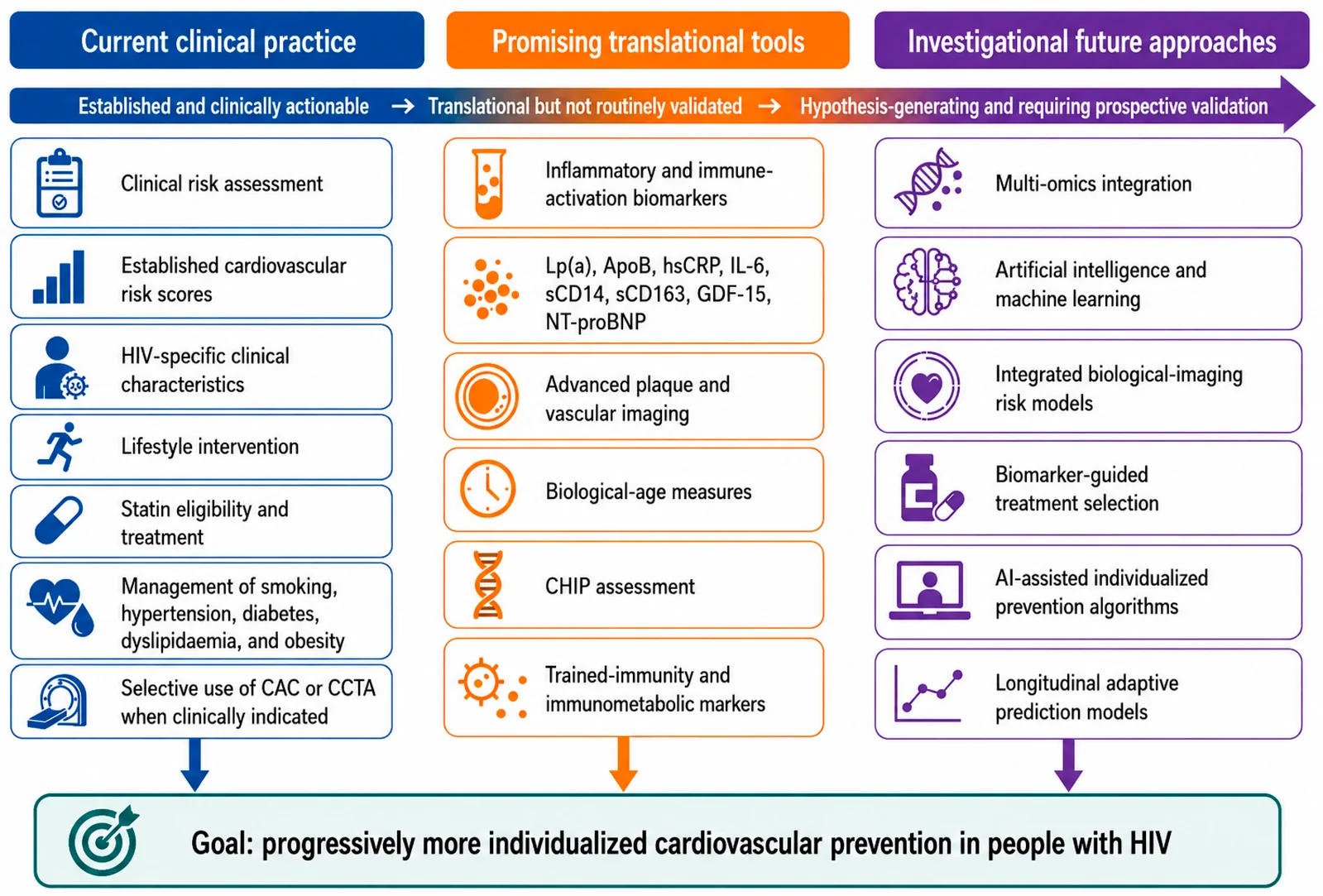
Precision cardiovascular prevention after REPRIEVE: current practice, translational tools, and investigational future approaches. The figure outlines three levels of maturity in cardiovascular prevention for people with HIV. Current clinical practice encompasses established, clinically actionable approaches: conventional cardiovascular risk assessment, validated risk scores, HIV-specific clinical characteristics, lifestyle interventions, statin eligibility and treatment, management of smoking, hypertension, diabetes, dyslipidemia, and obesity, and selective use of coronary artery calcium (CAC) scoring or coronary computed tomography angiography (CCTA) when clinically indicated. Promising translational tools include inflammatory and immune-activation biomarkers; lipid and cardiovascular biomarkers such as Lp(a), ApoB, hsCRP, IL-6, sCD14, sCD163, GDF-15, and NT-proBNP; advanced plaque and vascular imaging; biological-age measures; clonal hematopoiesis of indeterminate potential (CHIP) assessment; and trained-immunity and immunometabolic markers. These approaches are biologically and clinically promising but are not yet routinely validated for treatment selection. Investigational future approaches include multi-omics integration, artificial intelligence and machine learning, integrated biological-imaging risk models, biomarker-guided treatment selection, AI-assisted individualized prevention algorithms, and longitudinal adaptive prediction models. These remain hypothesis-generating and require prospective validation before routine implementation. The overarching goal is progressively more individualized cardiovascular prevention in people with HIV. This framework is conceptual and should not be interpreted as a validated clinical decision algorithm. **Abbreviations:** AI, artificial intelligence; ApoB, apolipoprotein B; CAC, coronary artery calcium; CCTA, coronary computed tomography angiography; CHIP, clonal hematopoiesis of indeterminate potential; GDF-15, growth differentiation factor-15; hsCRP, high-sensitivity C-reactive protein; IL-6, interleukin-6; Lp(a), lipoprotein(a); NT-proBNP, N-terminal pro-B-type natriuretic peptide; sCD14, soluble CD14; sCD163, soluble CD163.

These observations indicate that residual CVD reflects the interplay of multiple biological pathways rather than persistent dyslipidemia alone. Consequently, cardiovascular prevention after REPRIEVE increasingly requires integrating biomarkers, imaging, and biological profiling into individualized prevention strategies. Future research may determine whether combinations of emerging tools, including molecular profiling and multi-omics, improve cardiovascular risk assessment in PWH beyond conventional clinical models. Prospective studies should evaluate whether incorporating traditional risk factors alongside inflammatory biomarkers improves risk estimation and clinical decision-making. The framework proposed in Figure 3 is therefore conceptual and hypothesis-generating. It is intended to illustrate a possible direction for future research rather than a validated clinical pathway (see Figure 3).

## 6. Imaging, Multi-Omics, and Precision Cardiovascular Prevention

Given the limitations of conventional CVD prediction models in PWH, precision prevention strategies that integrate clinical characteristics, circulating biomarkers, and advanced cardiovascular imaging are increasingly being investigated to improve individualized CVD assessment and guide preventive interventions [5,11,12]. Modern research approaches aim to characterize the biological pathways contributing to atherosclerosis in individual patients, although their ability to improve event prediction or guide individualized prevention has not yet been established in PWH [25].

Among noninvasive imaging tests for assessing coronary artery disease in PWH, CCTA is the most robust test for several reasons. While CAC scoring, using non-contrast CT scans, provides an indicator of the amount of atherosclerotic plaque burden (i.e., hardened plaque), CCTA provides an assessment of the extent of atherosclerotic plaque (i.e., hardened and soft plaque), characterizes atherosclerotic changes in the arterial wall, identifies low-density plaque, and captures other features that are particularly relevant in PWH-associated CVD [13,17].

Coronary artery calcium scoring and carotid ultrasound offer complementary advantages. CAC scoring is standardized, widely available, relatively inexpensive, and useful for refining ASCVD risk, but it does not identify non-calcified coronary plaque, which may be particularly relevant in PWH [5,13]. Carotid ultrasound is radiation-free and accessible, and it can detect carotid plaque and measure intima–media thickness, although results are more operator-dependent and provide only an indirect assessment of coronary atherosclerosis [5]. By contrast, CCTA directly characterizes coronary plaque burden, composition, stenosis, and high-risk features, but it requires iodinated contrast, radiation exposure, greater technical expertise, and higher cost [5,13,17]. These modalities should therefore be considered complementary, with selection guided by the clinical question, local resources, and the likelihood that the findings will alter management.

Functional imaging of the vascular system can provide information beyond the structural characteristics of atherosclerotic plaques. Positron emission tomography (PET), especially when combined with CT or MRI, can be used to assess and quantify inflammatory processes within the vasculature [57]. Previous studies have identified increased vascular inflammation in PWH on effective ART, a finding supported by circulating immune-activation biomarkers (e.g., soluble CD163, IL-6) [11,57]. Other plaque features, such as composition and the presence of hemorrhage or a wall cavity, can be assessed using various MRI sequences. However, their use in clinical settings remains largely restricted to research [58].

Although CVD measures and imaging techniques provide information on the characteristics of atherogenesis in PWH, additional biomarkers and detailed molecular analyses are needed to adequately characterize it in these patients. To this end, a variety of omics technologies, such as transcriptomics, proteomics, metabolomics, lipidomics, and epigenomics, as well as single-cell analyses, are used. These technologies generate detailed biological profiles that may reveal mechanisms and potential therapeutic targets. However, their incremental predictive performance and clinical usefulness in PWH remain uncertain [59,60].

Recent advances in health information have enabled the integration of large amounts of clinical, imaging, laboratory, and molecular data using Artificial Intelligence (AI) and Machine Learning (ML) techniques. These techniques can simultaneously analyze multiple variables across different data types to uncover complex relationships that would otherwise go undetected. Early studies suggest that machine-learning methods may improve prediction in selected datasets, but external validation, assessment of bias, calibration, interpretability, and evidence of clinical benefit are required before routine use [61].

Despite their potential, AI and machine-learning models face substantial barriers to clinical implementation for PWH. Most available models have been developed in retrospective or selected cohorts and require external validation across independent populations, healthcare systems, geographic regions, and clinically diverse HIV populations. Assessing calibration, discrimination, transportability, and incremental value beyond established risk scores is essential. Limited interpretability may also reduce clinician confidence and make it difficult to identify erroneous or biologically implausible predictions. Data harmonization remains a major challenge because clinical variables, imaging protocols, biomarker platforms, and omics measurements differ across studies and institutions. In addition, algorithmic bias may arise when women, older adults, racial and ethnic minorities, people from low- and middle-income countries, individuals with uncontrolled HIV, and other underrepresented groups are inadequately represented in training datasets. Ethical issues related to privacy, informed consent, transparency, accountability, equitable access, and the risk of widening existing health disparities must also be addressed. Therefore, AI-based cardiovascular risk tools should remain investigational until they have undergone robust external validation and demonstrated fairness, interpretability, clinical utility, and improvement in patient outcomes.

AI and machine-learning methods may eventually complement current CVD scores, but they should be considered investigational tools for now. Therefore, multi-omics and computational approaches may help identify biological pathways and improve risk stratification, but their incremental predictive value and clinical utility in PWH remain to be established [59,60,61].

The recently validated PREVENT equations for predicting cardiovascular events in the general population represent an important advancement in the field, based on the largest population-based data set to date [75]. However, in the REPRIEVE study, PREVENT risk scores tended to underestimate the risk of cardiovascular events compared with earlier pooled-cohort equations [76]. Many individuals in the REPRIEVE study would have been classified as having low CVD and therefore not in need of statin therapy using PREVENT [76]. These important findings highlight a major limitation of PREVENT and other commonly used CVD scores for PWH and underscore the urgent need for HIV-specific CVD scores that have been validated in appropriate studies. Most of the adjunctive strategies discussed in this section have not been tested specifically for cardiovascular-event prevention in PWH. Their inclusion reflects biological plausibility and emerging evidence, not current recommendations for routine clinical use.

New methods for estimating CVD in PWH typically rely on a single test or factor and therefore often fail to account for the unique variables that determine risk in this population. It will be necessary to incorporate CVD tools alongside HIV-specific clinical data, measures of inflammation and immune activation, and the latest advances in imaging and molecular profiling to develop individualized risk models for CVD prevention. PWH are a highly biologically diverse population, and CVD likely results from the overlap of many factors that, in healthy individuals, would each independently increase CVD, regardless of cholesterol levels. As proposed in Figure 3**,** precision cardiovascular prevention should combine the four complementary domains previously outlined to estimate residual CVD more accurately than conventional prediction models [25].

## 7. Emerging Therapeutic Strategies Beyond Statins

Because residual cardiovascular risk persists despite ART and statin therapy, several adjunctive strategies are being investigated. Their rationale derives from the inflammatory, metabolic, and thrombotic pathways described above, but most have not been tested specifically for cardiovascular event prevention in PWH. Most of the adjunctive strategies discussed in this section have not been tested specifically for cardiovascular-event prevention in PWH. Their inclusion reflects biological plausibility and emerging evidence, not current recommendations for routine clinical use.

Anti-inflammatory therapy provides biological proof of concept in atherosclerosis, particularly through the IL-1β–IL-6 axis. However, CANTOS, COLCOT, and LoDoCo2 were not HIV-specific primary-prevention trials, and current evidence is insufficient to recommend routine anti-inflammatory therapy for cardiovascular prevention in PWH [62,77,78,79].

GLP-1RAs have emerged as promising interventions because of their broad cardiometabolic effects that extend beyond weight reduction and glycemic control. Large cardiovascular outcome trials in the general population have consistently shown reductions in major cardiovascular events among individuals with obesity and type 2 diabetes [70,71]. GLP-1RAs attenuate vascular inflammation, improve endothelial function, reduce oxidative stress, enhance insulin sensitivity, and modulate the biology of visceral adipose tissue. These mechanisms are particularly relevant to HIV-associated cardiovascular disease [72,73]. Although clinical experience with GLP-1RAs in PWH is currently limited [74], these properties provide a rationale for evaluating GLP-1 receptor agonists in future cardiovascular-prevention trials in PWH, but current evidence is insufficient to recommend them specifically for cardiovascular risk reduction in this population. Their role in HIV-associated cardiovascular prevention therefore requires dedicated prospective trials.

Lipoprotein(a) [Lp(a)] has emerged as a promising therapeutic target. Genetic and epidemiological evidence support Lp(a) as an independent risk factor and a likely causal contributor to atherosclerotic CVD [64]. Unlike LDL-C, Lp(a) levels are primarily genetically determined and minimally affected by statin therapy. Novel RNA-based therapies, such as antisense oligonucleotides and small interfering RNA targeting apolipoprotein(a), have demonstrated substantial reductions in circulating Lp(a) concentrations and are being evaluated in large cardiovascular outcome trials [65,66,67]. Although it remains unclear whether these agents confer specific benefits for PWH, elevated Lp(a) levels may help identify individuals with persistent CVD despite optimal statin therapy. Definitive cardiovascular outcome data are still emerging, and no HIV-specific outcome trials have been completed. Their relevance to PWH remains uncertain.

Interventions targeting trained immunity, AMPK, mTOR, mitochondrial dysfunction, or cellular senescence remain predominantly preclinical. Their safety, efficacy, and clinical relevance for cardiovascular prevention in PWH have not been established [11,63,80,81]. Although these approaches are primarily preclinical, they mark a shift from lipid-focused therapy toward modulation of the biological mechanisms that sustain persistent CVD in PWH.

CHIP is another area of active investigation. Recent analyses from the REPRIEVE trial indicate that large CHIP clones, particularly those involving DNMT3A, identify a subgroup of PWH at significantly increased risk of myocardial infarction and coronary revascularization [56]. Although no therapies currently target CHIP specifically for cardiovascular prevention, inhibition of downstream inflammatory pathways, especially interleukin-1β signaling, has been proposed as a biologically plausible strategy. Exploratory analyses suggest that inhibition of inflammatory pathways may have differential effects by CHIP genotype, including TET2-associated CHIP [82]. However, no therapy is currently established to prevent cardiovascular events specifically by targeting or eliminating mutant hematopoietic clones.

Taken together, these developments suggest that future cardiovascular prevention in PWH will extend beyond simply adding new lipid-lowering agents. Rather than replacing clinical judgment, AI should facilitate the integration of the multiple biological domains summarized in Figure 3, thereby improving individualized treatment selection. Thus, the key challenge will be to identify which patients are most likely to benefit from each intervention by integrating clinical characteristics, biomarkers, imaging, and molecular profiling within a precision medicine framework. Accordingly, these approaches should be considered priorities for translational and clinical research rather than therapies ready for routine implementation.

## 8. Conclusions

Despite effective ART, PWH continue to experience excess cardiovascular risk that is not fully explained by conventional risk factors. Persistent inflammatory, vascular, and metabolic abnormalities may contribute to this residual risk, but their individual clinical relevance varies, and many remain non-actionable.

REPRIEVE established pitavastatin as an effective primary-prevention intervention for eligible PWH and showed that residual cardiovascular risk remains. Current care should therefore remain anchored in statin use when indicated and in intensive management of established risk factors. Biomarkers, advanced imaging, biological-age measures, CHIP, multi-omics, AI, and emerging therapies may refine prevention in the future, but most remain translational or investigational and require prospective validation.

After REPRIEVE, there has been a shift in how cardiovascular prevention is viewed for PWH. An important research priority is to determine whether statins can be combined with validated biological and imaging tools to improve risk stratification and prevention beyond current standards. Ongoing research should aim to confirm the value of precision prevention methods that integrate standard risk assessments, HIV-specific clinical details, biomarkers, advanced imaging, and molecular analyses. It is also important to test new therapies that target residual inflammation and metabolic risks. This broad strategy holds the greatest promise for reducing the burden of heart disease among PWH. At present, precision cardiovascular prevention in PWH should primarily mean the individualized application of validated preventive measures; broader integration of biomarkers, multi-omics, advanced imaging, and AI remains investigational. Until such strategies are prospectively validated, clinical management should remain anchored in evidence-based statin use and intensive control of established CVD factors.

## Data Availability

No new data were created or analyzed in this study. Data sharing is not applicable to this article.

## Abbreviations

AI	Artificial intelligence
AMPK	AMP-activated protein kinase
ApoB	Apolipoprotein B
ART	Antiretroviral therapy
ASCVD	Atherosclerotic cardiovascular disease
CAC	Coronary artery calcium
CANTOS	Canakinumab Anti-inflammatory Thrombosis Outcomes Study
CHIP	Clonal hematopoiesis of indeterminate potential
CCTA	Coronary computed tomography angiography
CT	Computed tomography
CVD	Cardiovascular disease
D:A:D	Data Collection on Adverse Events of Anti-HIV Drugs
DNA	Deoxyribonucleic acid
DNMT3A	DNA methyltransferase 3 alpha
eNOS	Endothelial nitric oxide synthase
GLP-1-RA	Glucagon-like peptide-1 receptor agonist
GDF-15	Growth differentiation factor-15
HIF-1α	Hypoxia-inducible factor-1 alpha
HIV	Human immunodeficiency virus
HLA-DR	Human leukocyte antigen-DR
HMG-CoA	3-Hydroxy-3-methylglutaryl-coenzyme A
hsCRP	High-sensitivity C-reactive protein
ICAM-1	Intercellular adhesion molecule-1
IFN-γ	Interferon-gamma
IL	Interleukin
IP-10	Interferon gamma-induced protein 10
LDL-C	Low-density lipoprotein cholesterol
LMICs	Low- and middle-income countries
Lp(a)	Lipoprotein(a)
LPS	Lipopolysaccharide
MACE	Major adverse cardiovascular events
MCP-1	Monocyte chemoattractant protein-1
MMPs	Matrix metalloproteinases
MRI	Magnetic resonance imaging
mTOR	Mammalian target of rapamycin
mtDNA	Mitochondrial DNA
NADPH	Nicotinamide adenine dinucleotide phosphate
NF-κB	Nuclear factor kappa B
NLRP3	NOD-like receptor family pyrin domain-containing 3
NO	Nitric oxide
non-HDL-C	Non-high-density lipoprotein cholesterol
NT-proBNP	N-terminal pro-B-type natriuretic peptide
oxLDL	Oxidized low-density lipoprotein
OXPHOS	Oxidative phosphorylation
PAI-1	Plasminogen activator inhibitor-1
PAMPs	Pathogen-associated molecular patterns
PET	Positron emission tomography
PREVENT	Prediction of Risk of Cardiovascular Events
PWH	People with HIV
REPRIEVE	Randomized Trial to Prevent Vascular Events in HIV
RNA	Ribonucleic acid
ROS	Reactive oxygen species
SCORE2	Systematic Coronary Risk Evaluation 2
sCD14	Soluble CD14
sCD163	Soluble CD163
TNF-α	Tumor necrosis factor-alpha
Tregs	Regulatory T cells
VCAM-1	Vascular cell adhesion molecule-1
